# Ultrasmall PEI-Decorated Bi_2_Se_3_ Nanodots as a Multifunctional Theranostic Nanoplatform for *in vivo* CT Imaging-Guided Cancer Photothermal Therapy

**DOI:** 10.3389/fphar.2021.795012

**Published:** 2021-12-02

**Authors:** Peng Zhang, Lei Wang, Xiuying Chen, Xiang Li, Qinghai Yuan

**Affiliations:** ^1^ Department of Radiology, The Second Hospital of Jilin University, Changchun, China; ^2^ Department of Radiology, Jilin Province FAW General Hospital, Changchun, China

**Keywords:** photothermal therapy, Bi2Se3 nanodots, ultrafast synthesis, CT imaging, cancer therapy

## Abstract

Bi-based nanomaterials, such as Bi_2_Se_3_, play an important part in biomedicine, such as photothermal therapy (PTT) and computed tomography (CT) imaging. Polyethylenimine (PEI)-modified ultrasmall Bi_2_Se_3_ nanodots were prepared using an ultrafast synthetic method at room temperature (25°C). Bi_2_Se_3_ nanodots exhibited superior CT imaging performance, and could be used as effective photothermal reagents owing to their broad absorption in the ultraviolet–visible–near infrared region. Under irradiation at 808 nm, PEI-Bi_2_Se_3_ nanodots exhibited excellent photothermal-conversion efficiency of up to 41.3%. Good biocompatibility and significant tumor-ablation capabilities were demonstrated *in vitro* and *in vivo*. These results revealed that PEI-Bi_2_Se_3_ nanodots are safe and a good nanotheranostic platform for CT imaging-guided PTT of cancer.

## Introduction

The early diagnosis of cancer and “precision” medicine are major challenges for oncologists. Scientists need to develop multifunctional biocompatible nanotheranostic platforms that integrate diagnostic and therapeutic functions (Barreto et al., 2011; Ho et al., 2015; Chen et al., 2016). As a non-invasive method, phototherapy can alleviate the side-effects and suffering of treatment as compared with that using resection, chemotherapy, or radiotherapy (Cheng et al., 2014; Fan et al., 2017; Lei et al., 2018; Basak et al., 2019).

Photothermal therapy (PTT) has attracted significant research attention because it is highly efficient, minimally invasive, and controllable (Liu et al., 2019; Meng et al., 2020; Zhao et al., 2020). Recently, PTT agents, such as precious metals (e.g., Au, Pt, or Pd nanoparticles) (Yin et al., 2014; Tang et al., 2015; Zhu et al., 2017; Zhang et al., 2019), metal chalcogenides (e.g., CuS nanoparticles, Bi_2_S_3_ nanoparticles, Bi_2_Se_3_ nanosheets, MoS_2_ nanosheets, or MoSe_2_ nanosheets) (Liu et al., 2014; Yang et al., 2016; You et al., 2017; Huang et al., 2019; Wang et al., 2019), carbon derivatives (Bao et al., 2018; Ortega-Liebana et al., 2019), and polymeric nanoparticles (Han et al., 2018; Zhang et al., 2018), have aroused widespread research interest. In particular, Bi_2_Se_3_, with its broad near absorption in the infrared (NIR) region, excellent efficiency for photothermal conversion, good biocompatibility, and metabolizability, has been used as a PTT agent (Song et al., 2015; Cheng et al., 2016; Xie et al., 2017; Huang et al., 2019). Moreover, due to the high X-ray attenuation coefficient (5.74 cm^2^ g^−1^, 100 keV) and atomic number (Z = 83) of Bi than those of the extensively applied contrast agent iobitridol (X-ray attenuation coefficient of 1.94 cm^2^ g^−1^, 100 keV; Z = 53), Bi-based nanomaterials can be used as potential contrast agents for computed tomography (CT) (Lei et al., 2017). Thus, Bi_2_Se_3_ has been used widely as a powerful nanotheranostic agent in CT imaging-guided PTT of cancer. However, most reports have focused on the synthesis of Bi_2_Se_3_ nanosheets at high temperatures (Xiao et al., 2017; Xie et al., 2017). Nevertheless, the synthesis is more complicated, and a larger particle size is not conducive to biological metabolism. Only a few reports have focused on the ultrarapid synthesis of water-soluble ultrasmall Bi_2_Se_3_ nanodots at room temperature (25°C).

We report a facile room-temperature method for synthesizing ultrasmall polyethylenimine-decorated Bi_2_Se_3_ (PEI-Bi_2_Se_3_) nanodots for CT imaging-guided PTT of cancer *in vitro* and *in vivo*. Compared with strategies reported previously, the rapid synthesis of PEI-Bi_2_Se_3_ nanodots may improve the efficiency of the synthesis and prevent further surface modification. Furthermore, the raw materials are inexpensive, and the organic solvents are nontoxic and environmentally friendly, thereby making the process suitable for future production at a large scale.

The synthesized nanodots exhibited excellent absorption properties and the efficiency of photothermal conversion was high under laser irradiation at 808 nm. Moreover, the outstanding CT imaging and photothermal-ablation capacity observed *in vitro* and *in vivo*, and non-significant long-term toxicity observed *in vivo*, revealed that PEI-Bi_2_Se_3_ nanodots could achieve CT imaging-guided PTT of cancer. Hence, PEI-Bi2Se3 nanodots could be powerful and safe nanotheranostic agents in cancer therapy.

## Results and Discussion

### The Synthesis and Characterization of PEI-Bi_2_Se_3_ Nanodots

**SCHEME 1:**
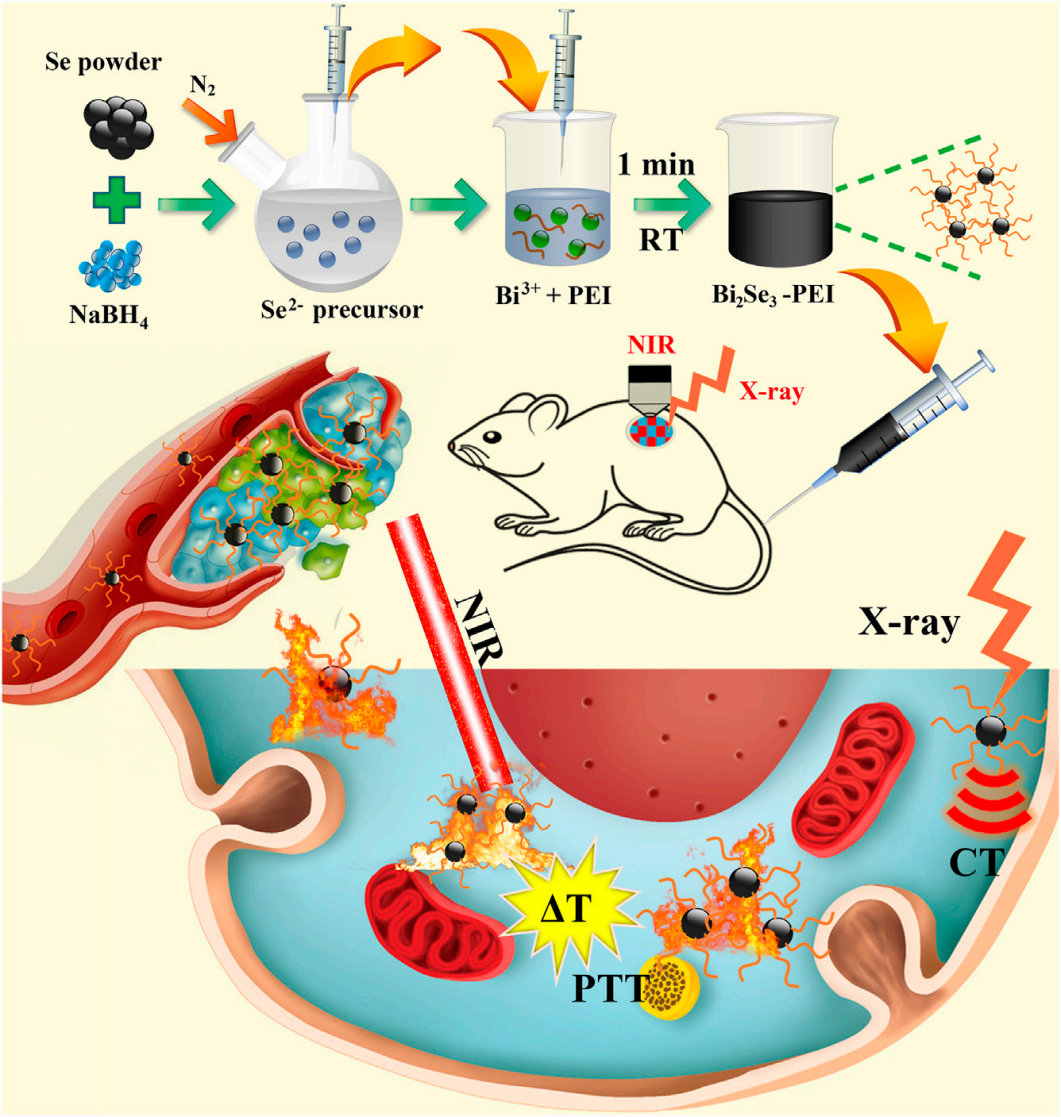
Fabrication of PEI-Bi_2_Se_3_ nanodots for CT imaging-guided PTT of cancer.

A novel NIR light-responsive nanotheranostic platform based on PEI-Bi_2_Se_3_ nanodots for CT imaging-guided PTT of cancer was fabricated (Scheme 1). Under the protection of an inert-gas atmosphere and ice-water bath, Se powder was reduced by NaBH_4_ under magnetic stirring at room temperature (25°C) to obtain NaHSe solution (Se^2−^ precursor solution). An appropriate amount of the Se^2−^ precursor solution was transferred rapidly to a mixed solution of ethylene glycol and water containing Bi(NO_3_)_3_·5H_2_O and PEI. The reaction solution turned black rapidly, indicating that the reaction was ultra-facile and efficient (within 1 min). In the Experimental section in Supplementary Material, the experimental details are presented. Moreover, Bi_2_Se_3_ nanodots had promising potential for CT imaging-guided PTT of cancer owing to an efficient photothermal performance, strong absorption in the NIR region, and the high X-ray attenuation coefficient of Bi^3+^.

**FIGURE 1:**
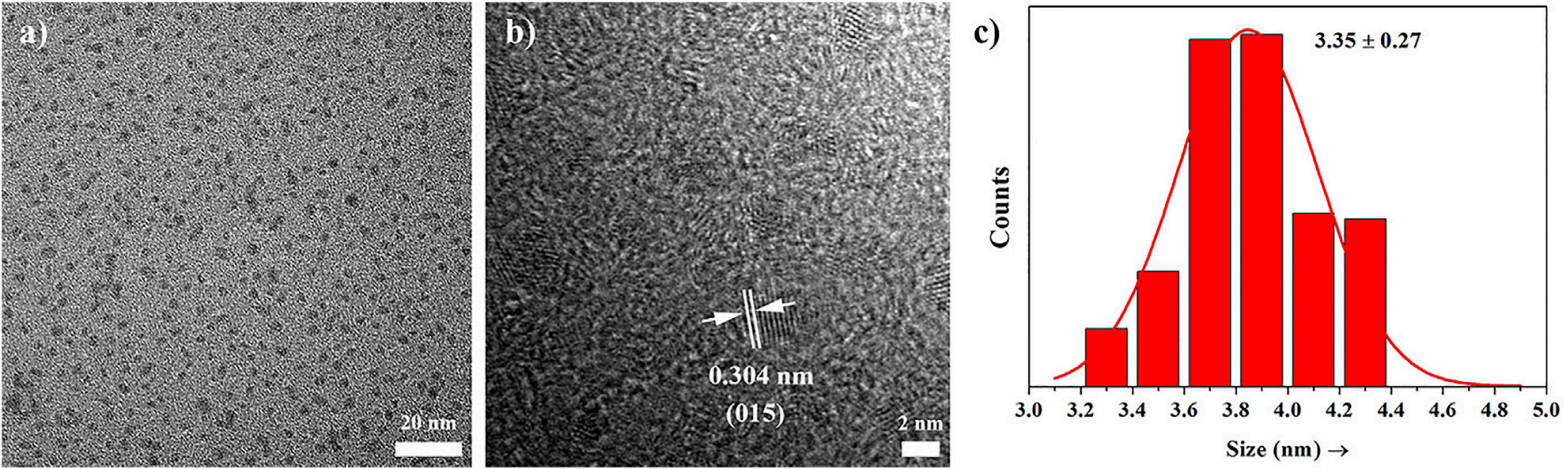
**(A)** TEM image, **(B)** HRTEM image, and **(C)** size histogram of PEI-Bi_2_Se_3_ nanodots.

A transmission electron microscopy (TEM) image of the obtained PEI-Bi_2_Se_3_ nanodots is shown in Figure 1A. A clear lattice fringe with a distance of 0.304 nm can be seen on the high-resolution TEM image (Figure 1B), which can be attributed to the (015) planes of Bi_2_Se_3._ Furthermore, the prepared PEI-Bi_2_Se_3_ nanodots, as uniform spheres with relatively narrow size distribution, had a mean diameter of 3.56 nm (Figure 1C). The X-ray diffraction (XRD) patterns of the prepared PEI-Bi_2_Se_3_ nanodots are shown in Supplementary Figure S1. All the characteristic XRD peaks matched well with the standard hexagonal phase of Bi_2_Se_3_ (Joint Committee on Powder Diffraction Standards = 33-0214). Moreover, PEI-Bi_2_Se_3_ nanodots could maintain good dispersity in various solutions, such as Dulbecco’s modified Eagle’s medium, phosphate-buffered saline (PBS), NaCl, and water, for several months (Supplementary Figure S2), indicating that the PEI modification was beneficial for improving the stability of PEI-Bi_2_Se_3_ nanodots.

### Photothermal Performance of PEI-Bi_2_Se_3_ Nanodots *in vitro*

**FIGURE 2:**
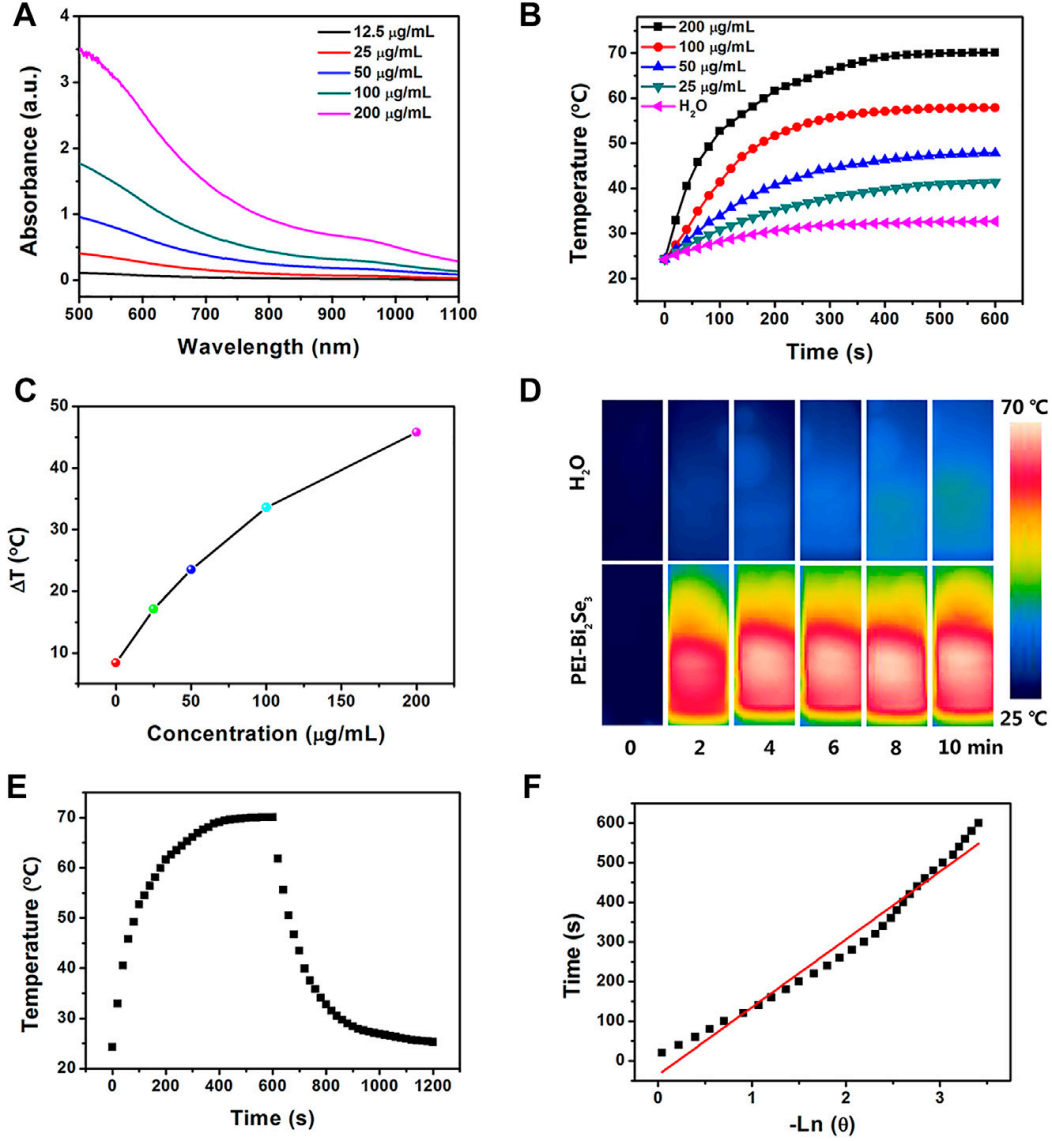
**(A)** UV–Vis–NIR absorption spectra of PEI-Bi_2_Se_3_ nanodots in water with different concentrations of Bi^+3^. **(B)** Plot of temperature increase of pure water and aqueous solutions of PEI-Bi_2_Se_3_ nanodots of different concentrations upon exposure to a NIR laser at 808 nm (1.0 Wcm^−2^) as a function of irradiation duration. The temperature was measured every 10 s using a thermocouple microprobe. **(C)** Plot of temperature change (Δ*T*) over a period of 600 s vs. the concentration of PEI-Bi_2_Se_3_ nanodots. **(D)** IR thermal images of an aqueous solution of PEI-Bi_2_Se_3_ nanodots (200 μg ml^−1^) and pure water after irradiation for 10 min (808 nm, 1.0 W cm^−2^). **(E)** Photothermal response of an aqueous solution of PEI-Bi_2_Se_3_ nanodots (200 μg ml^−1^) after irradiation for 600 s with an NIR laser (808 nm, 1.0 W cm^−2^). Subsequently, the laser was turned off. **(F)** Linear time data vs. −lnθ obtained after a cooling period of **(E)**.

PEI-Bi_2_Se_3_ nanodots showed a broad ultraviolet–visible–NIR (UV–Vis–NIR) absorption spectrum ranging from 500 to 1,100 nm (Figure 2A). As the concentration of Bi^3+^ increased, the absorption intensity of PEI-Bi_2_Se_3_ nanodots was enhanced, and the colorless solution turned dark black (Supplementary Figure S3). Absorbance at 808 nm increased linearly (Supplementary Figure S4), which suggested that PEI-Bi_2_Se_3_ nanodots exhibited good dispersibility in water, and could be excellent photothermal agents for PTT. To investigate the photothermal performance, pure water (control) and aqueous solutions of PEI-Bi_2_Se_3_ nanodots (25, 50, 100, 200 μg/ml) were exposed to a NIR laser (808 nm, 1.0 W cm^−2^) for 10 min. The temperatures of the different PEI-Bi_2_Se_3_ nanodot solutions increased rapidly, exhibiting noticeable concentration- and irradiation time-dependent behavior (Figure 2B). This finding indicated that the temperature of the PEI-Bi_2_Se_3_ solution could reach up to 70.1°C at a concentration of 200 μg ml^−1^ (1.0 W cm^−2^, 10 min), which is highly effective for killing tumor cells *via* hyperthermia. In contrast, under identical experimental conditions, the temperature of pure water increased by up to 8.4°C (Figure 2C). The IR thermal images of pure water and aqueous solution of PEI-Bi_2_Se_3_ nanodots (200 μg ml^−1^) after a certain duration of irradiation are shown in Figure 2D. The temperature of the PEI-Bi_2_Se_3_ aqueous solution (200 μg ml^−1^) increased rapidly with an increase in the irradiation duration using a laser at 808 nm whereas, under identical conditions, the temperature of pure water increased slowly.

PEI-Bi_2_Se_3_ nanodots could therefore convert NIR energy into thermal energy rapidly and efficiently, and act as potential photothermal agents during PTT. In particular, the efficiency of photothermal conversion of PEI-Bi_2_Se_3_ nanodots was up to 41.3% (Figures 2E,F), which is much higher than that of currently reported photothermal agents, such as PVP-Bi nanodots (∼30%) (Lei et al., 2017), Bi_2_Se_3_ nanosheets (∼33%) (Xie et al., 2017), and Cu_2−*x*_Se nanocrystals (∼22%) (Hessel et al., 2011). Photostability is another prerequisite for evaluating the performance of photothermal agents during PTT. After irradiation of the aqueous solution of PEI-Bi_2_Se_3_ nanodots (200 μg ml^−1^) using a continuous-wave NIR laser at 808 nm for 1 h (1.0 W cm^−2^), the color of the solution, UV–Vis–NIR spectrum, and morphology exhibited no distinct changes (Supplementary Figures S5, S6). Hence, PEI-Bi_2_Se_3_ nanodots possessed satisfactory photothermal stability. All the results shown above (excellent photothermal effect and good photostability) highlighted the potential of PEI-Bi_2_Se_3_ nanodots as suitable agents for PTT of cancer.

### Studies on Cytotoxicity and Photothermal Ablation of Tumor Cells

**FIGURE 3:**
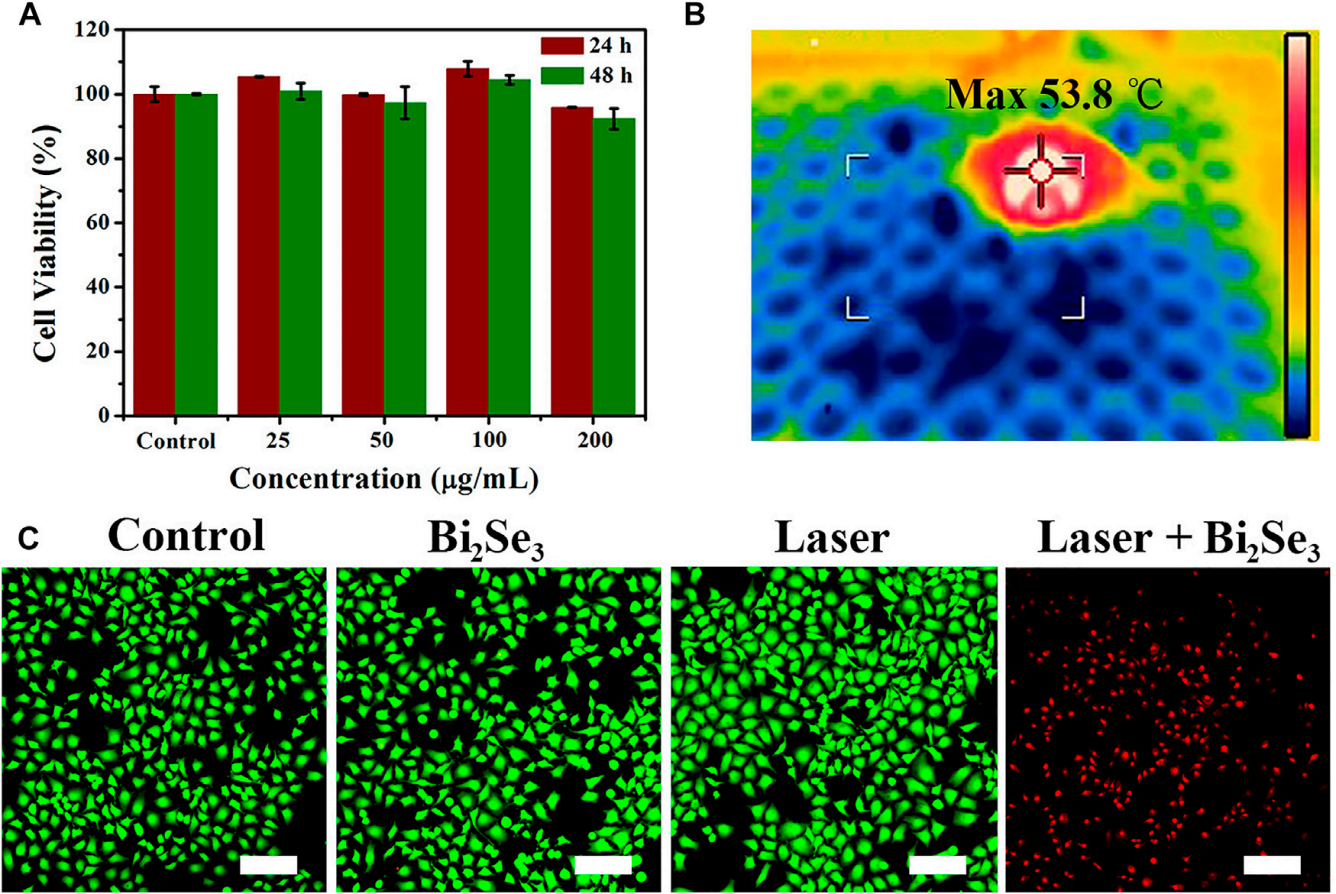
**(A)** Viability of A549 cells incubated with PEI-Bi_2_Se_3_ nanodots (0–200 μg ml^−1^) for 24 and 48 h **(B)** IR thermal images of A549 cells in a 96-well plate incubated with PEI-Bi_2_Se_3_ nanodots after irradiation for 10 min (808 nm, 1.0 W cm^−2^). **(C)** Fluorescence images of A549 cells stained with calcein-AM (live cells, green) and PI (dead cells, red) after various treatments. Scale bar = 200 μm.

Evaluation of the cytotoxicity of PEI-Bi_2_Se_3_ nanodots is important. The cytotoxicity of PEI-Bi_2_Se_3_ nanodots was tested by the Cell Counting Kit-8 assay. Even at a high concentration of PEI-Bi_2_Se_3_ nanodots (200 μg ml^−1^), the viability of A549 cells was 96 and 92% after incubation for 24 h (red bars) and 48 h (green bars), respectively (Figure 3A). These results indicated that PEI-Bi_2_Se_3_ nanodots exhibited no distinct toxicity towards A549 cells.

Because of the outstanding photothermal performance of PEI-Bi_2_Se_3_ nanodots, we investigated their photothermal effects against tumor cells. The IR thermal images of A549 cells incubated with PEI-Bi_2_Se_3_ nanodots in a 96-well plate are shown in Figure 3B. Notably, the temperature could increase up to 53.8°C under irradiation at 808 nm (1.0 W cm^−2^) after addition of PEI-Bi_2_Se_3_ nanodots to the culture. Cancer cells are sensitive to heat, and can be killed effectively at >42°C. To identify further the anti-cancer effect of PEI-Bi_2_Se_3_ nanodots on A549 cells, live and dead cells were imaged using a fluorescence microscope after staining with calcein acetoxymethyl ester (green fluorescence) and propidium iodide (red fluorescence), respectively. In the control groups, notable cytotoxicity was not observed (PBS, PEI-Bi_2_Se_3_ nanodots only, laser only), whereas almost no living cells were observed in the PEI-Bi_2_Se_3_ + laser group (Figure 3C). These results suggested that the as-synthesized PEI-Bi_2_Se_3_ nanodots with low cytotoxicity would produce satisfactory results upon *in vivo* cancer treatment. In addition, endocytosis pathways were determined in order to identify the uptake mechanism of extracellular PEI-Bi_2_Se_3_ nanodots. The cellular uptake of PEI-Bi_2_Se_3_ was evaluated by monitoring the fluorescence of FITC in the A549 cells at various incubation times (1, 3, and 6 h). As shown in Supplementary Figure S7, green fluorescence of FITC was observed after 1 h incubation with FITC-labeled PEI-Bi_2_Se_3_. The signals increase obviously with incubation time, demonstrating efficient internalization of PEI-Bi_2_Se_3_ by cancer cells.

### CT Imaging *in vitro* and *in vivo*

**FIGURE 4:**
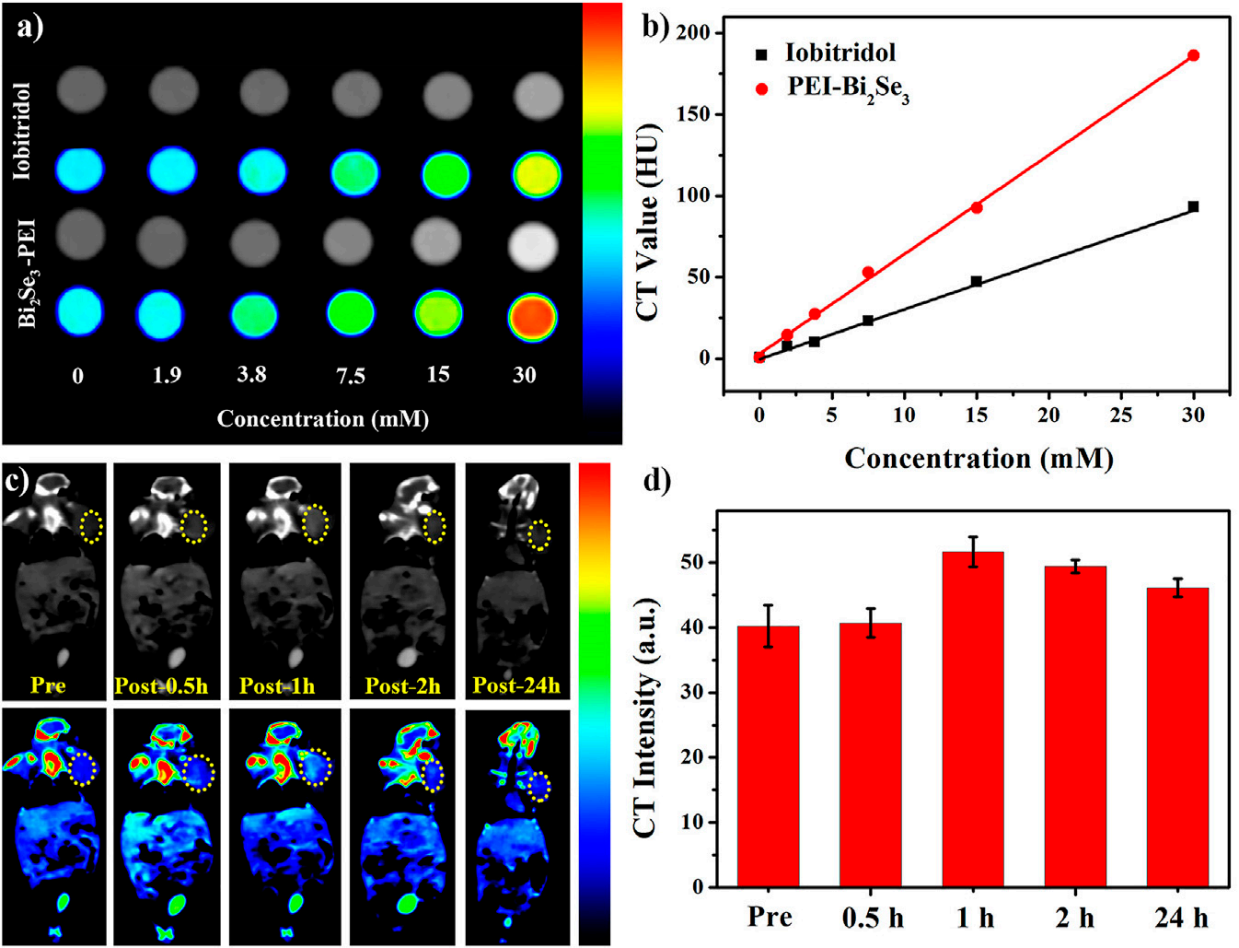
**(A)** *In vitro* CT images of iobitridol and PEI-Bi_2_Se_3_ nanodots at different concentrations. **(B)** Concentration-dependent CT signals of iobitridol (black line) and PEI-Bi_2_Se_3_ nanodots (red line) *in vitro*. **(C)** Time-dependent CT imaging of tumor-bearing mice before and after intravenous injection of PEI-Bi_2_Se_3_ nanodots. **(D)** CT signal intensities of the tumor area at different times.

High resolution, easy manipulation, and high penetrability make CT imaging an important part of medical diagnoses (Lee et al., 2013; Du et al., 2020). Bi element with its large atomic number and high electron density has promising capacity for X-ray attenuation (Kinsella et al., 2011; Li et al., 2016). The X-ray absorption coefficient and iobitridol were compared to evaluate the *in vitro* CT imaging capability of PEI-Bi_2_Se_3_ nanodots. The latter exhibited a much higher CT density than that of iobitridol at equivalent concentrations (Figure 4A), whereas the Hounsfield unit (HU) values of both contrast agents exhibited a typical linear dependence on the concentration (Figure 4B). Compared with the curve for iobitridol, the curve for PEI-Bi_2_Se_3_ nanodots had a steeper slope. Hence, the as-synthesized PEI-Bi_2_Se_3_ nanodots had superior ability in CT imaging and were effective contrast agents.

Inspired by the satisfactory CT effect *in vitro*, we assessed the feasibility of using PEI-Bi_2_Se_3_ nanodots as CT contrast agents *in vivo*. Time-dependent CT imaging was undertaken after tumor-bearing mice were injected (i.v.) with PEI-Bi_2_Se_3_ nanodots (Bi concentration = 30 mM, 150 μL). At 0 h–1 h after injection, the CT density at the tumor site brightened gradually (Figure 4C), which was caused by passive accumulation of PEI-Bi_2_Se_3_ nanodots at the tumor site through the enhanced permeability and retention effect. Thereafter, the density decreased because some PEI-Bi_2_Se_3_ nanodots had metabolized. The mean HU value increased from 40.2 HU (pre-injection) to 40.7 HU (0.5 h) and 51.6 HU (1 h) and decreased gradually from 49.4 HU (2 h) to 46.1 HU (24 h) at the tumor site (Figure 4D). These results demonstrated that PEI-Bi_2_Se_3_ nanodots could serve as promising *in vivo* CT contrast agents for the accurate diagnosis of cancer.

### Photothermal Effect, Photothermal Therapy, and Long-Term Toxicity of PEI-Bi_2_Se_3_ Nanodots *in vivo*

**FIGURE 5:**
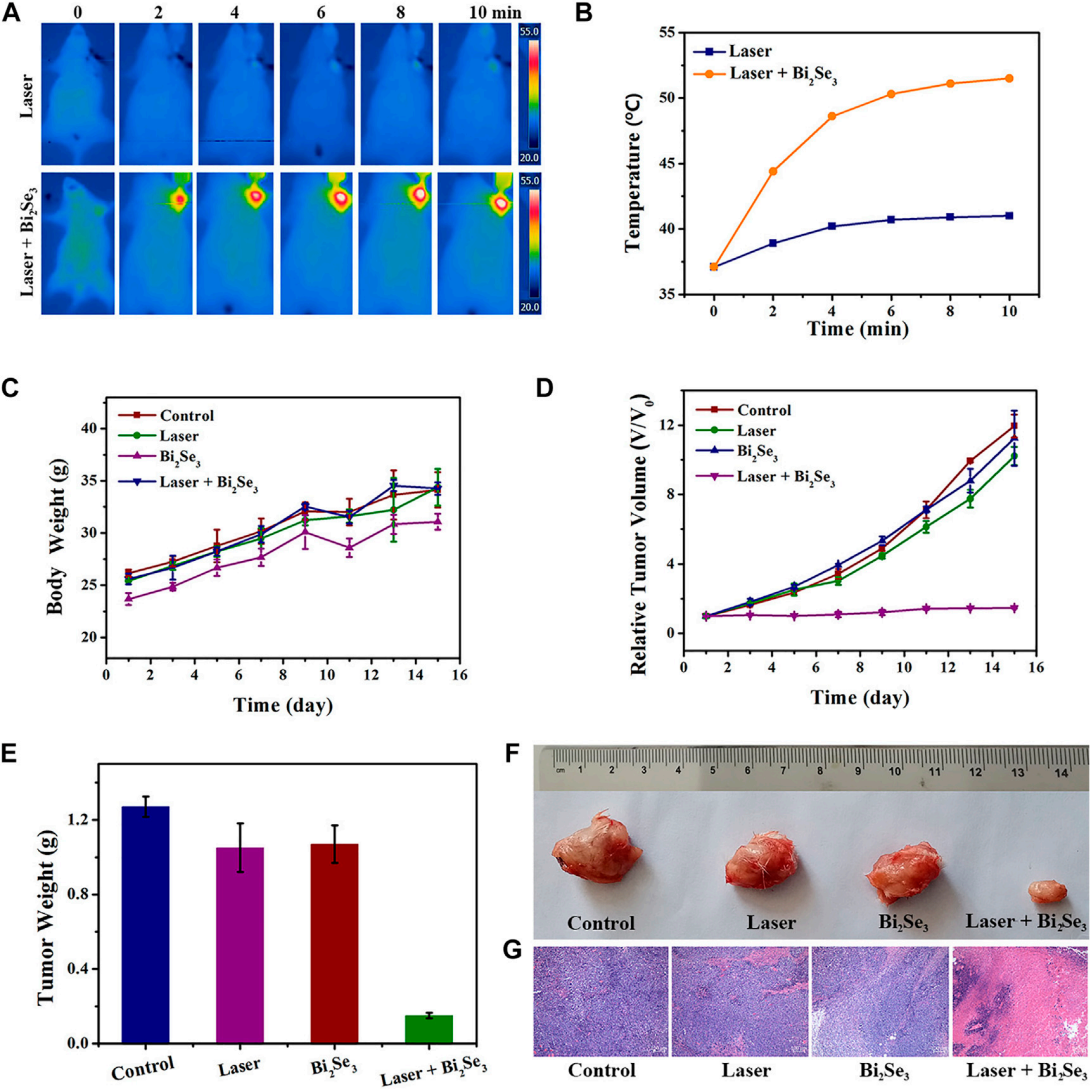
**(A)** IR thermal images and **(B)** corresponding curve showing the temperature variation of tumor-bearing mice injected (i.v.) with PBS (control) or PEI-Bi_2_Se_3_ nanodots, followed by irradiation with a laser at 808 nm for 10 min **(C)** Bodyweight of mice and **(D)** curves showing relative tumor growth in different groups after various treatments. **(E)** Mean tumor weight of each group after various treatments. **(F)** Photographs of tumors of each group and **(G)** H&E staining of tumor slides collected from different groups. All scale bars = 100 µm.

Based on the good properties of PEI-Bi_2_Se_3_ nanodots at tumor sites (i.e., excellent *in vitro* photothermal effect, satisfactory CT imaging effect, and outstanding passive targeted accumulation), we studied the feasibility of CT imaging-guided PTT of cancer *in vivo*. PEI-Bi_2_Se_3_ nanodots (Bi concentration = 20 mg kg^−1^) were injected (i.v.) into tumor-bearing mice. Obvious enhancement was observed 1 h after injection, so the tumor was irradiated using a laser at 808 nm (1.0 W cm^−2^) 1-h later, and the photothermal effect *in vivo* was monitored by an IR thermal camera. The temperature at the tumor site in the treatment group increased rapidly as the duration of irradiation increased (Figure 5A) but the temperature at the tumor site did not show a significant change compared with that in the control group. The corresponding curve detailing temperature variation is shown as Figure 5B. After 10 min of NIR irradiation (808 nm, 1.0 W cm^−2^), in the presence of PEI-Bi_2_Se_3_ nanodots, the temperature at the tumor sites was as high as 51.1°C whereas, in the control group, a minor increase in temperature was observed. Hence, PEI-Bi_2_Se_3_ nanodots could serve as excellent photothermal agents for *in vivo* tumor ablation: they could kill cancer cells and inhibit their continued diffusion.

A tumor model was established by injecting (s.c.) U14 cells into the left axilla of female Kunming mice. Once the tumors had grown to ∼100 mm^3^, mice were used for experimentation. The mice bearing the U14 cells were divided randomly into four groups of six: 1) control; 2) laser only; 3) PEI-Bi_2_Se_3_ nanodots; 4) PEI-Bi_2_Se_3_ nanodots + laser. The tumors in mice were irradiated (808 nm) 1 h after injection of PEI-Bi_2_Se_3_ nanodots. The bodyweight and tumor volume of mice were measured every 2 days to evaluate therapeutic efficacy. After various treatments, the bodyweight of mice showed a steady increase (Figure 5C), thereby indicating that PEI-Bi_2_Se_3_ nanodots did not produce toxic side-effects during PTT. The tumor volume of each mouse was measured using a Vernier caliper, and plotted as a function of the relative tumor volume and treatment duration (Figure 5D). The average weights of excised tumors are shown in Figure 5E, and representative tumor photographs of each group are shown in Figure 5F. Compared with groups 1–3, the growth of tumors in group 4 was inhibited significantly after 14 days of PTT. In addition, hematoxylin and eosin (H&E) staining revealed no appreciable damage in groups 1–3; simultaneously, severe shrinkage and discrete cancer cells were observed clearly in group 4 (Figure 5G). Taken together, these results demonstrated that PEI-Bi_2_Se_3_ nanodots possessed potential as ideal and safe photothermal agents for cancer treatment.

**FIGURE 6:**
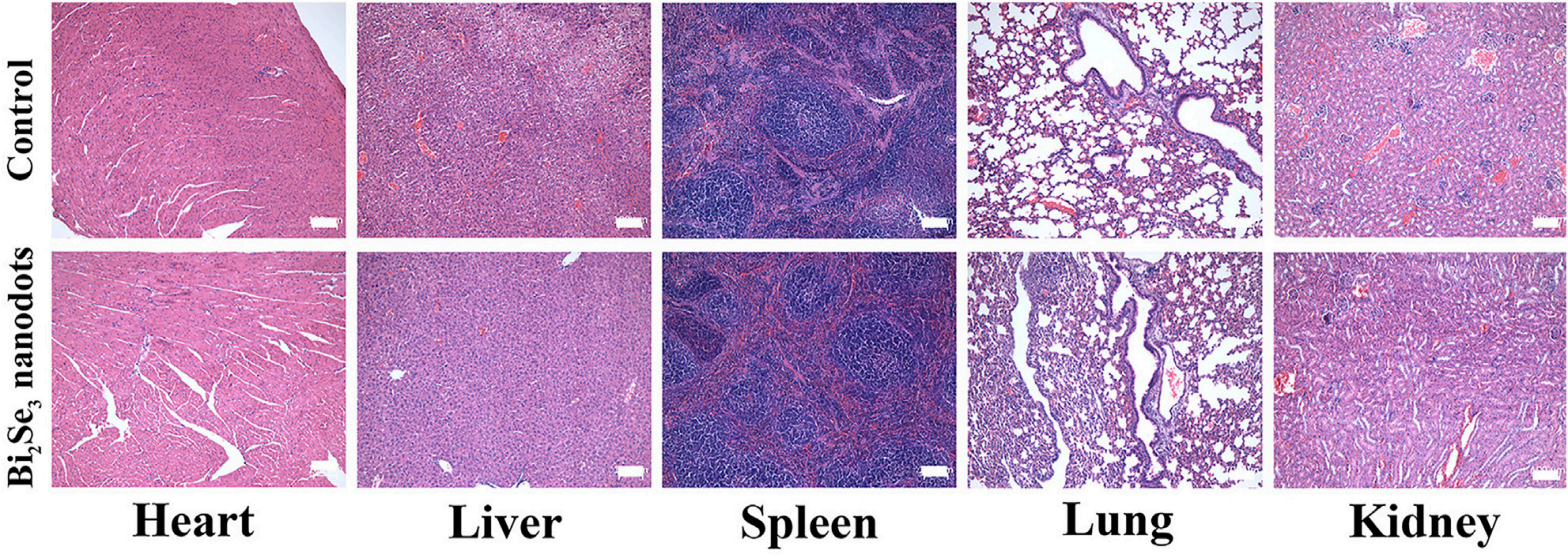
H&E staining of major organs (heart, liver, spleen, lungs, and kidneys) of mice 30 days after injection of PEI-Bi_2_Se_3_ nanodots. All scale bars = 100 µm.

The potential long-term toxicity of PEI-Bi_2_Se_3_ nanodots *in vivo* was also investigated. Thirty days after injection, pathological samples of major organs (heart, lungs, liver, spleen, kidneys) from control mice and treated mice were obtained. H&E staining (Figure 6) revealed no distinct tissue damage or inflammatory lesions in any major organ. Moreover, there were no abnormal signs in treated mice during the entire observation period. These results confirmed that PEI-Bi_2_Se_3_ nanodots were not significantly toxic *in vivo*.

## Conclusion

Ultrasmall PEI-Bi_2_Se_3_ nanodots were fabricated *via* an ultrafast, facile, and environmentally friendly method. The obtained PEI-Bi_2_Se_3_ nanodots could ensure good contrast enhancement owing to their high X-ray attenuation coefficient, and showed good photothermal killing effects *in vitro* and *in vivo* owing to considerable photothermal-conversion effects. Moreover, PEI-Bi_2_Se_3_ nanodots possessed negligible long-term toxicity *in vivo*. Therefore, we believe that the as-synthesized PEI-Bi_2_Se_3_ nanodots are useful theranostic agents for CT-imaging-guided PTT of cancer.

## Data Availability

The original contributions presented in the study are included in the article/Supplementary Material, further inquiries can be directed to the corresponding author.
