# Supplementary material for: Ultrasmall PEI-Decorated Bi2Se3 Nanodots as a Multifunctional Theranostic Nanoplatform for in vivo CT Imaging-Guided Cancer Photothermal Therapy
Source: Front Pharmacol. 2021 Dec 2;12:795012. doi: 10.3389/fphar.2021.795012 (PMC8675356; doi:10.3389/fphar.2021.795012)
Supplement: Supplementary file 1 [file DataSheet1.docx]

Supplementary Material

# Experimental section

## Materials

PEI (molecular weight = 10,000 g mol^-1^, 99%), bismuth nitrate pentahydrate (Bi(NO_3_)_3_·5H_2_O, 99%), and Se powder were purchased from Aladdin Reagents Co., Ltd. (Shanghai, China). Ethylene glycol (EG) and NaBH_4_ were obtained from Beijing Chemical Reagent Materials Co. (Beijing, China). All chemical reagents were of analytical grade, and used without further purification.

## Synthesis of PEI-Bi_2_Se_3_ nanodots

In a typical procedure, 100 mg of PEI was dissolved in a mixture of 3 mL of water and 14 mL of ethanol at room temperature (25℃) under vigorous stirring. Thereafter, 60.6 mg of Bi(NO_3_)_3_ was dissolved in a 2 mL mixture of water and EG (1:1 ratio) (solution A). The Se powder (236.85 mg) was reduced using NaBH_4_ (243.3 mg) in 5 mL deionized water under magnetic stirring at room temperature (25℃) under the protection of an inert gas and ice-water bath (NaHSe solution, 0.6 mol/L, solution B). Finally, 0.12 mmol of solution B was directly injected into solution A, and stirred for 1 min. After the completion of the reaction, the resulting nanodots were dialyzed (molecular weight cut-off = 20,000) against deionized water for 24 h.

## Characterization

Powder XRD patterns were recorded using a Bruker D8 ADVANCE X-ray diffractometer with Cu Kα radiation (l = 1.5418 Å), while the operating voltage and current were maintained at 40 kV and 40 mA, respectively. Low-resolution TEM/HRTEM were performed using an FEI TECNAI G2 high-resolution transmission electron microscope operating at 200 kV. The UV–Vis–NIR absorption spectra were measured using a Shimadzu UV-3600 spectrophotometer (Shimadzu Co., Japan). Inductively coupled plasma (ICP) optical emission spectroscopy was performed using an iCAP 6000 ICP emission spectrometer (Thermo Fisher Scientific). ICP mass spectrometry was performed on the Thermo Fisher X series^II^.

## Cytotoxicity assay of PEI-Bi_2_Se_3_ nanodots

Typical CCK-8 assays were performed to assess the cytotoxicity of the PEI-Bi_2_Se_3_ nanodots. The A549 cells were first placed in a 96-well plate (approximately 5000 per well), and cultured at 37 °C with 5% CO_2_ for 24 h in DMEM, supplemented with 10% fetal bovine serum. Thereafter, the cells were washed with PBS, and incubated with the PEI-Bi_2_Se_3_ nanodots with different Bi concentrations (0, 25, 50, 100, and 200 μg/mL) at 37 °C for 24 h, and then, for an additional 24 h (total of 48 h). Subsequently, the cell viability was detected via CCK-8 assay. The absorbance of each solution was measured at 450 nm using a microplate reader (Varioskan Flash, Thermo Fisher Scientific).

## In Vitro Cellular Uptake

Firstly, the synthesized PEI-Bi_2_Se_3_ nanodots was labeled by fluorescein isothiocyanate (FITC) by the reaction between the isothiocyanate groups of FITC with the NH_2_ group of PEI. Briefly, FITC dissolved in DMSO (1 mg mL^-1^, 1 mL) was mixed 8 mL PEI-Bi_2_Se_3_ aqueous solution. Then, 1 mL Na_2_CO_3_ solution (0.5 M) was added and the mixture was stirred at room temperature in dark for 24 h. FITC labeled PEI-Bi_2_Se_3_ was obtained by dialyzing (molecular weight cut off =14000) in water for 48 h. Finally, in vitro cellular uptake of the as-synthesized PEI-Bi_2_Se_3_ was evaluated by confocal laser scanning microscopy (CLSM) after co-culturing cancer cells and PEI-Bi_2_Se_3_ nanomaterial for various times (1, 3, 6 h).

## Animal experiments

Kunming mice were purchased from the Laboratory Animal Center of Jilin University (Changchun, China). Animal care and handling procedures were followed in accordance with the guidelines of the Regional Ethics Committee for Animal Experiments. The tumor models were established via subcutaneous injection of U14 cells into the left axilla of each mouse. The mice were used for experiments once the tumors had grown to a size of approximately 100 mm^3^.

## In vitro and in vivo X-ray CT imaging

To assess CT contrast efficacy, PEI-Bi_2_Se_3_ nanodots and iobitridol were dispersed in deionized water with different concentrations (0, 1.9, 3.8, 7.5, 15, and 30) of Bi and I, respectively. For *in vivo* CT imaging, the tumor-bearing mice were anesthetized with an intraperitoneal injection (100 μL) of 10% chloral hydrate. Subsequently, the PEI-Bi_2_Se_3_ nanodots were intravenously injected (Bi concentration = 30 mM, 150 μL) for CT imaging. The *in vitro* and *in vivo* CT images were acquired using a Philips 256-slice CT scanner. The imaging parameters were as follows: 120 kVp, 300 mA; thickness, 0.9 mm; pitch, 0.99; field of view, 350 mm; gantry rotation time, 0.5 s; and table speed, 158.9 mm s^−1^.

## Photothermal effect of PEI-Bi_2_Se_3_ nanodots

To determine the photothermal effect of the nanocomposites, 400 μL of aqueous solutions of the PEI-Bi_2_Se_3_ nanodots with different Bi concentrations (0, 25, 50, 100, and 200 μg mL^-1^) were exposed to the NIR laser (808 nm, 1.0 W cm^-2^) for 10 min. The solution temperature was measured every 10 s using a thermocouple microprobe. To evaluate the photothermal conversion efficiency (*η*), the aqueous solution of the PEI-Bi_2_Se_3_ nanodots (200 μg mL^-1^, 400 μL) was irradiated using the 808 nm laser with a power density of 1.0 W cm^-2^, until the temperature was steady. Thereafter, the laser was turned off, and the system temperature was naturally cooled to room temperature (25℃), which was determined by measuring the temperature every 10 s. The value of *η* was calculated according to the methods provided in previous literature.^1^

The photothermal conversion efficiency (*η*) was calculated by using equations (1)-(4):

*η* = hS (T_max,NPs_ – T_max,solvent_) / I (1 – 10^-A808^ ) (1)

*τ_s_* = m_d_C_d_ / hs (2)

t = -*τ_s_lnθ* (3)

*θ = T – T_surr_ /* (*T_max,_* _NPs_ *– T_max,solvent_)* (4)

where h is the heat transfer coefficient, S is the surface area of the container, *T_max,_*_NPs_ and *T_max,solvent_* are maximum steady-state temperature for PEI-Bi_2_Se_3_ nanodots solution and water, which are 70.1 and 32.7 °C for 808 nm laser, respectively. I is the incident laser power (1.0 W/cm^2^), and A_808_ is the absorbance of PEI-Bi_2_Se_3_ nanodots at 808 nm (A_808_ = 0.90). τ_s_ is the sample system time constant, and m_d_ and C_d_ are the mass (1.0 g) and heat capacity (4.2 J/g) of the deionized water used as the solvent, respectively. *θ* is the dimensionless driving force temperature, T_surr_ is the ambient temperature of the surroundings, T is a temperature for PEI-Bi_2_Se_3_ aqueous solutions at a constant cooling time (t), the τ_s,808_ was determined to be 173.88 s (Figure 2f). The photothermal conversion efficiency of PEI-Bi_2_Se_3_ nanodots was calculated according to equation (5):

*η* = m_d_C_d_ (*T_max,_*_NPs_ – T*_max,H2O_*) /I(1 – 10^-A808^ ) *τ_s_* (5).

## In vitro photothermal therapy of cancer cells

To study the photothermal cytotoxicity of PEI-Bi_2_Se_3_ nanodots, the cell live/dead assays were carried out to evaluate the efficiency of the PTT. Briefly, A549 cells seeded into a 24-well plate were divided into four groups (1−4) as follows: (1) control group (PBS solution), (2) NIR laser only group (808 nm, 1.0 W cm^-2^, 6 min), (3) PEI-Bi_2_Se_3_ group (Bi^3+^ = 200 μg mL^-1^), and (4)PEI-Bi_2_Se_3_ (Bi^3+^ = 200 μg mL^-1^) plus NIR laser group (808 nm, 1.0 W cm^-2^, 6 min). A mixed solution containing 2 μM of calcein AM and 8 μM of PI was then added to the wells. After being stained for 40 min, cells were washed with PBS and examined by using a fluorescence microscope to detect the live/dead status.

## In vivo photothermal effect

For *in vivo* photothermal imaging, the tumor-bearing mice were intravenously injected with the PEI-Bi_2_Se_3_ nanodots (Bi concentration = 20 mg kg^-1^). After 1 h, the tumor sites were exposed to the 808 nm laser (1.0 W cm^-2^, 6 min). During the NIR irradiation, an IR thermal camera was used to monitor the temperature changes at the tumor sites.

## In vivo photothermal therapy assays

When the tumor volume reached approximately 100 mm^3^, the tumor-bearing mice were randomly divided into four groups (n = 6) as follows: (1) control (PBS solution), (2) NIR laser only (808 nm, 1.0 W cm^-2^, 6 min), (3) PEI-Bi_2_Se_3_ (20 mg kg^-1^ Bi), and (4) PEI-Bi_2_Se_3_ (20 mg kg^-1^ Bi) plus NIR laser (808 nm, 1.0 W cm^-2^, 6 min). During the NIR irradiation, an IR thermal camera was used to monitor the temperature changes at the tumor sites. The tumor size and body weight of the mice before and after treatment were measured using a Vernier caliper and an electronic balance, respectively. The tumor volume was calculated as volume = width^2^ × length/2. The relative tumor volume was calculated as *V*/*V*_0_, where *V*_0_ is the corresponding tumor volume before treatment. After 14 days, to evaluate the therapeutic efficacy, the tumors were dissected and weighed.

## Histology analysis

For the *in vivo* toxicity studies, healthy Kunming mice were injected with 100 μL of the PEI-Bi_2_Se_3_ nanodots with a dosage of 20 mg kg^-1^ Bi. The mice that did not undergo any treatment were used as the blank control. Over a period of 30 days, the mice were sacrificed, and the main organs of the mice (heart, liver, spleen, lung, and kidney) were harvested and fixed using 4% paraformaldehyde. The tissue samples were then embedded in paraffin, sliced, and stained with H&E. The histological sections were observed using an optical microscope.


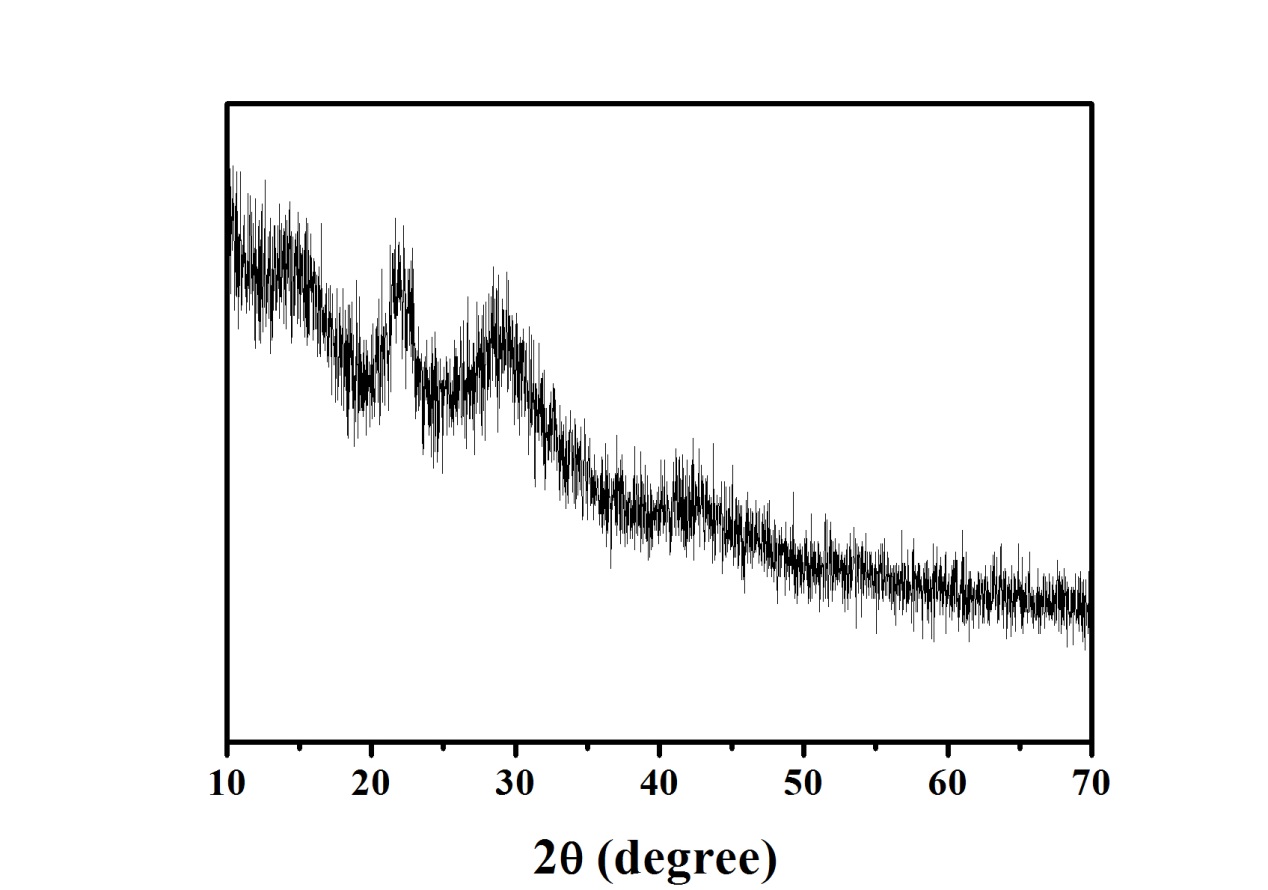


**Figure S1.** XRD patterns of PEI-Bi_2_Se_3_ nanodots.


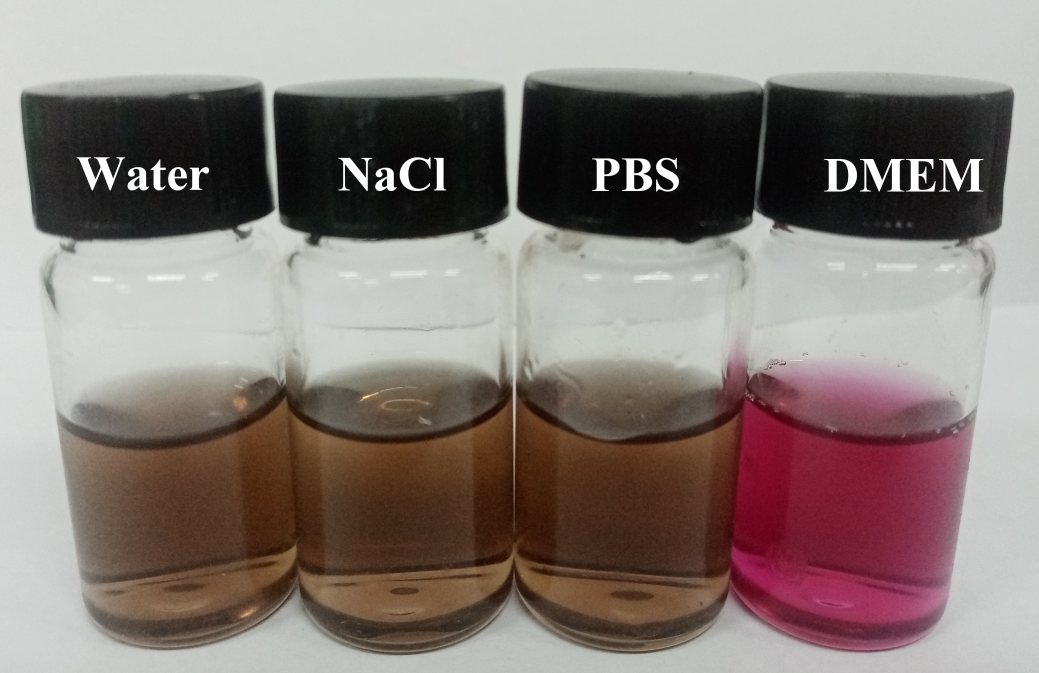


**Figure S2.** The digital photograph of PEI-Bi_2_Se_3_ nanodots in various solutions such as water, sodium chloride (NaCl), phosphate buffer saline (PBS), and Dulbecco’s modified eagle medium (DMEM) biological buffers for there months (Bi concentrations = 50 μg/mL).


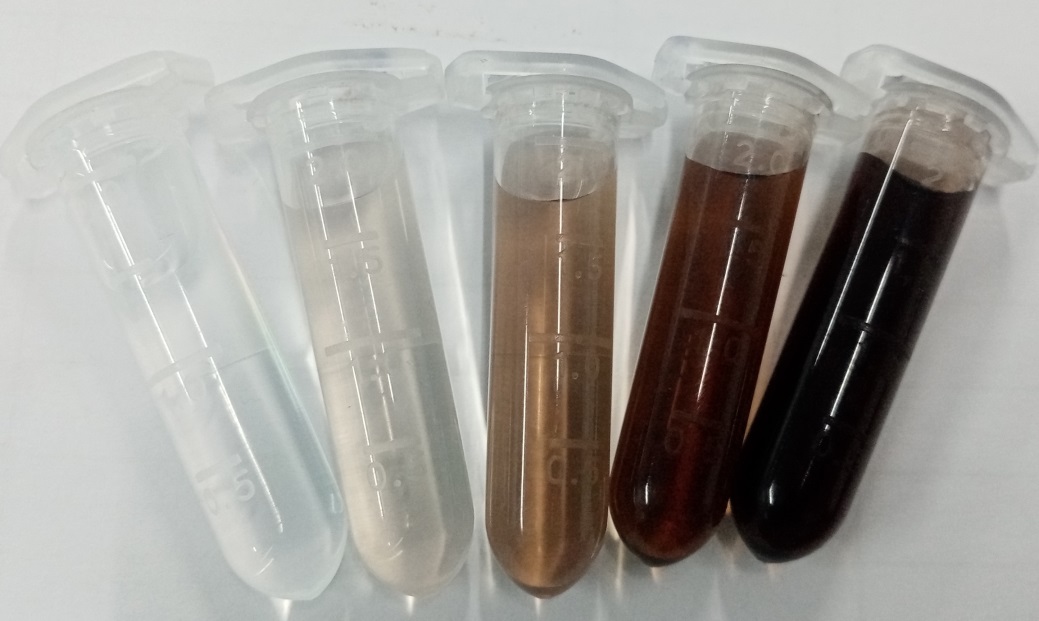


**Figure S3.** The digital photograph of PEI-Bi_2_Se_3_ nanodots aqueous solution with different concentrations (12.5, 25, 50, 100, and 200 μg/mL).


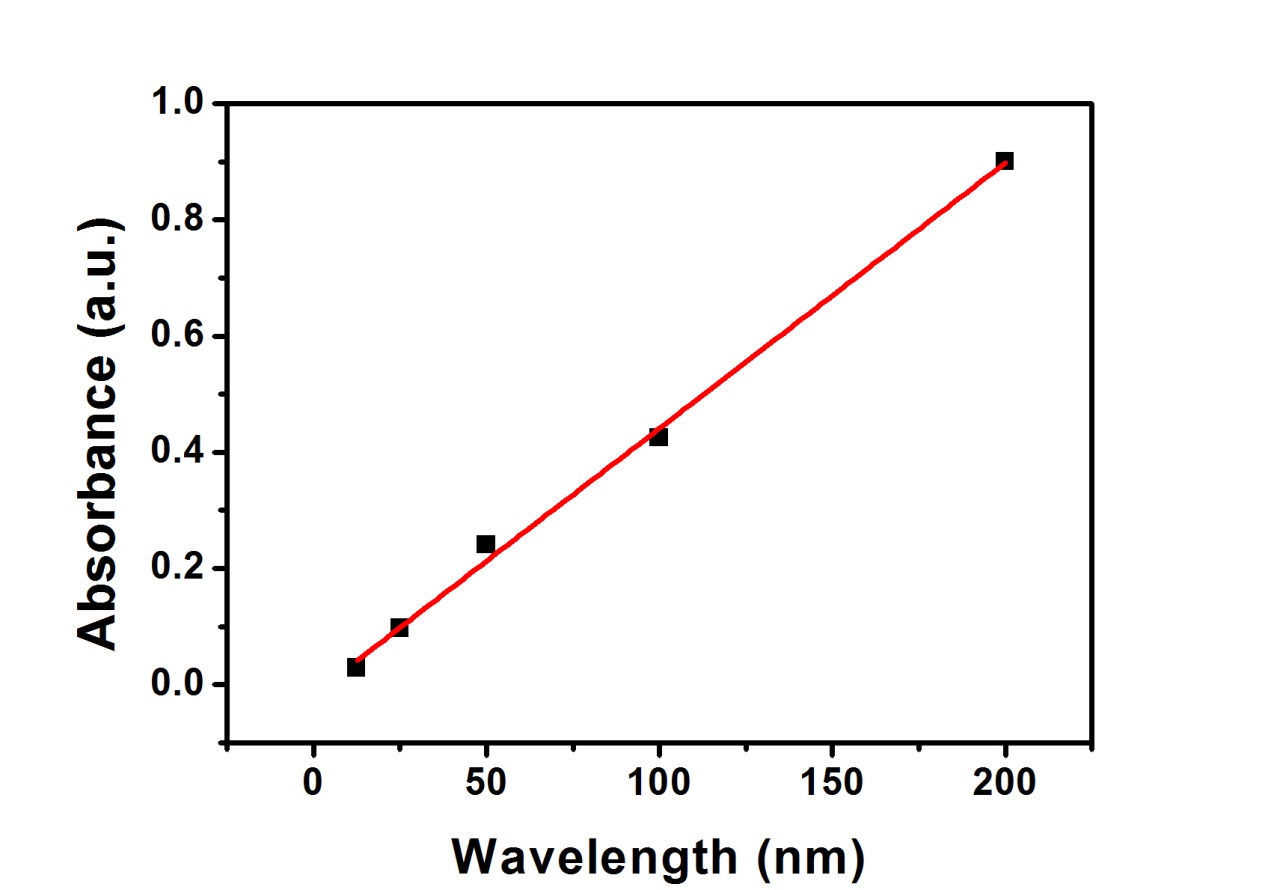


**Figure S4.** A linear relationship for the optical absorbance at 808 nm as a function of the concentration of PEI-Bi_2_Se_3_ nanodots.


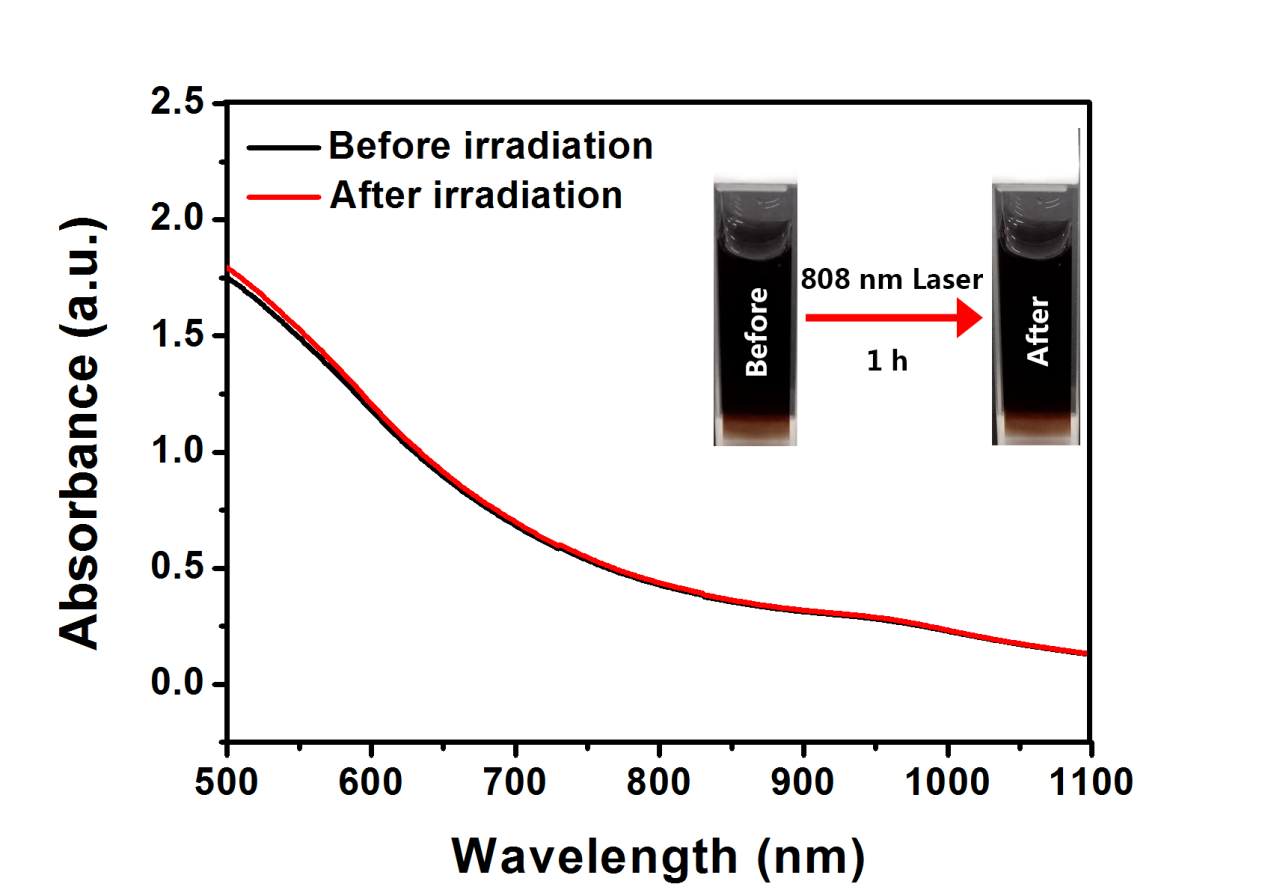


**Figure S5.** UV-vis-NIR absorption spectra of PEI-Bi_2_Se_3_ aqueous solution (Bi concentrations = 100 μg/mL) before and after 1 h laser irradiation (808 nm, 1.0 W cm^-2^); inset pictures represent the well-dispersed PEI-Bi_2_Se_3_ solution before and after irradiation.





**Figure S6.** TEM image of PEI-Bi_2_Se_3_ nanodots after 1 h laser irradiation (808 nm, 1.0 W cm^-2^).


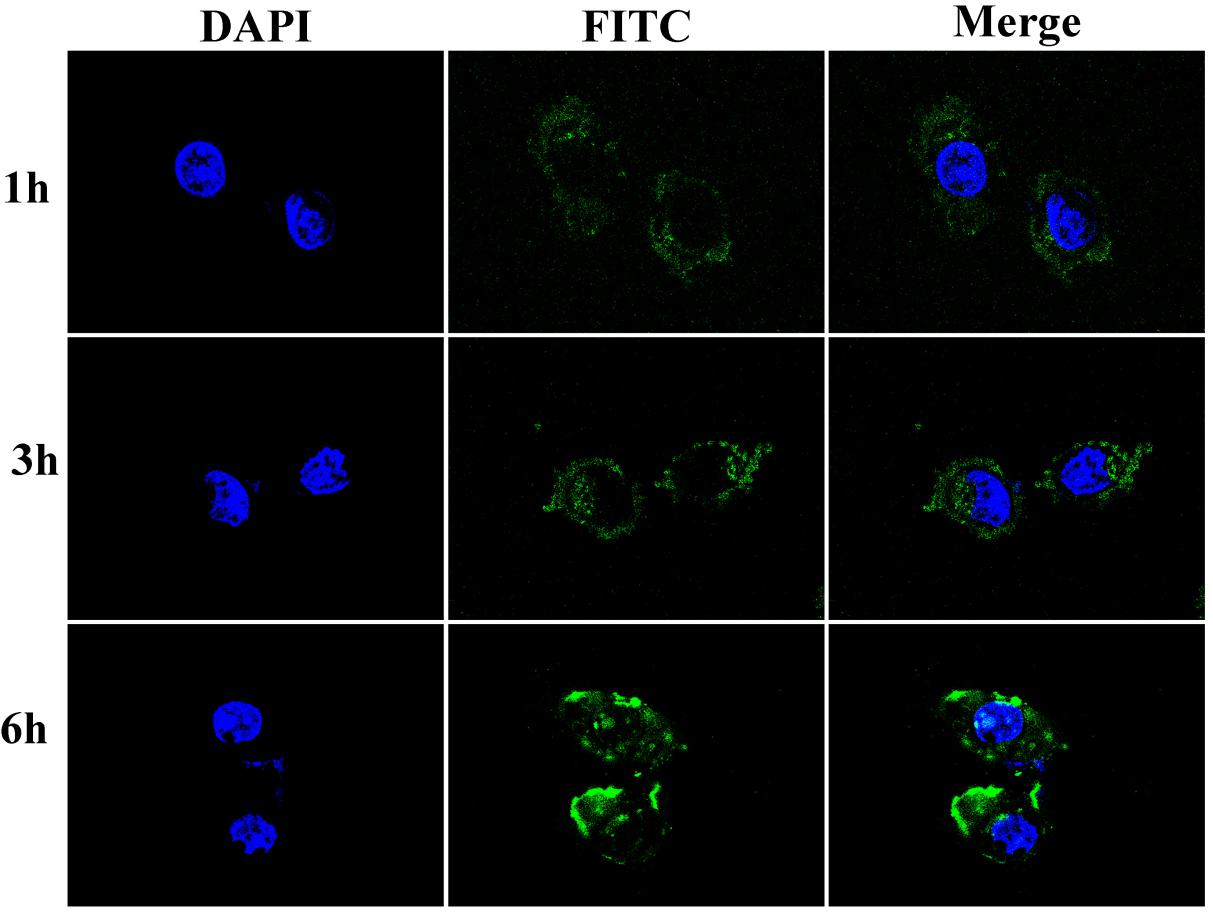


**Figure S7.** Confocal microscopic images of the intracellular distribution of FITC-labeled PEI-Bi_2_Se_3_ in A549 cells at various incubation times (1, 3, and 6 h).

1. Zhu, H.; Wang, Y.; Chen, C.; Ma, M.; Zeng, J.; Li, S.; Xia, Y.; Gao, M., Monodisperse Dual Plasmonic Au@Cu_2-x_E (E=S, Se) Core@Shell Supraparticles: Aqueous Fabrication, Multimodal Imaging, and Tumor Therapy at in Vivo Level. *ACS Nano* **2017,** *11* (8), 8273-8281.
